# Prolonged Suppression of Neuropathic Hypersensitivity upon Neurostimulation of the Posterior Insula in Mice

**DOI:** 10.3390/cells11203303

**Published:** 2022-10-20

**Authors:** Han Li, Zheng Gan, Lirong Wang, Manfred Josef Oswald, Rohini Kuner

**Affiliations:** Pharmacology Institute, Heidelberg University, Im Neuenheimer Feld 366, 69120 Heidelberg, Germany

**Keywords:** neuropathic pain, transcranial direct current stimulation, pain relief

## Abstract

Neurostimulation-based therapeutic approaches are emerging as alternatives to pharmacological drugs, but need further development to optimize efficacy and reduce variability. Despite its key relevance to pain, the insular cortex has not been explored in cortical neurostimulation approaches. Here, we developed an approach to perform repetitive transcranial direct current stimulation of the posterior insula (PI tDCS) and studied its impact on sensory and aversive components of neuropathic pain and pain-related anxiety and the underlying neural circuitry in mice using behavioral methods, pharmacological interventions and the expression of the activity-induced gene product, Fos. We observed that repetitive PI tDCS strongly attenuates the development of neuropathic mechanical allodynia and also reverses chronically established mechanical and cold allodynia for several weeks post-treatment by employing descending opioidergic antinociceptive pathways. Pain-related anxiety, but not pain-related aversion, were inhibited by PI tDCS. These effects were associated with a long-term suppression in the activity of key areas involved in pain modulation, such as the cingulate, prefrontal and motor cortices. These data uncover the significant potential of targeting the insular cortex with the objective of pain relief and open the way for more detailed mechanistic analyses that will contribute to improving cortical neurostimulation therapies for use in the clinical management of pain.

## 1. Introduction

Pain is a multidimensional experience, which remains a major challenge in terms of understanding fundamental mechanisms as well as therapeutic management. Chronic neuropathic pain is particularly refractory to therapy, and conventional pharmacological treatments are limited by side effects [1]. The role of cortical plasticity in positive or negative modulation of pain is paramount [2,3]. Therefore, tapping into cortical circuits and modulating their participation and plasticity in chronic pain holds therapeutic promise.

Specificity in modulating neocortical circuitry is difficult to reach with pharmacological agents since they distribute broadly and act at multiple loci. These drawbacks can be counterbalanced with neurostimulation approaches applied transcranially, such as transcranial magnetic stimulation (TMS) or transcranial direct current stimulation (tDCS) [4,5]. Indeed, transcranial cortical stimulation is emerging as a promising therapeutic approach for refractory neuropathic pain in clinical studies [6,7,8]. Despite this promise, there remain a number of concerns arising from major variations in efficacy, lack of insights into mechanistic underpinnings, and the need for optimizing the locus and regimens of neurostimulation. Studies in animal models can contribute to clarifying these important questions. 

A majority of previous studies investigating neurostimulation approaches in human subjects and rodent models have focused on the motor cortex [5,7,8,9,10], and a few new studies have also emerged on the prefrontal cortex [11]. In contrast, the insular cortex has not been tested so far in neurostimulation studies in rodents, and to date there is no knowledge on the potential of therapies specifically targeting the insula in achieving pain relief. This is remarkable, since the insula is one of the few brain areas in which ictal discharges have been reported to directly elicit the perception of pain, while lesions of the posterior insula impair nociceptive functions, although systematic and controlled studies are lacking [12]. A functional dichotomy has been suggested, with the posterior insular cortex participating in the somatosensory (nociceptive) features of pain and the anterior insula, preferably being involved in the affective dimensions of pain [13,14]. Recent studies on imaging and electrophysiological recordings in the human insula demonstrate strong activation of the posterior insula in thermal and mechanical nociception [15,16]. In addition to acute pain, the posterior insula has been implicated in chronic pain, with remarkable grey matter alterations being reported that are reversed upon adequate pain therapy. In models of rodent models of chronic pain, synapses in the insular cortex have been demonstrated to undergo functional plasticity [17]. Taken together, there is ample basis to warrant analyses on targeting the insular cortex for achieving pain control. 

Here, by employing direct current stimulation on the posterior insula, we sought to address whether insular stimulation positively or negatively modulates neuropathic pain in mice. Importantly, with a view towards enhancing translational promise, we tested the implications of repetitive cycles of stimulation and sought to address the cellular mechanistic basis of changes in brain excitation following insular direct current stimulation.

## 2. Materials and Methods

### 2.1. Animals

All experiments were conducted in C57BL/6J mice (20–30 g) of both sexes at 8 weeks of age that were obtained from Janvier Labs. In total, 31 animals were used and the sex ratio was balanced across all experiments. Mice were housed individually in separated cages and kept under a 12 h light/dark cycle at a controlled temperature (22 ± 2 °C), humidity (40–50%), and with food and water provided ad libitum according to ARRIVE guidelines. All experimental procedures were approved by the local governing body (Regierungspräsidium Karlsruhe, Germany, Ref. 35-9185.81/G-184/18, 35-9185.81/G-205/14), and was in accordance with the German law that regulates animal welfare and the protection of animals used for scientific purposes (TierSchG, TierSchVersV).

### 2.2. Experimental Design

Mice were allowed to recover for 3–6 days following electrode implantation. Mice were randomly divided into two groups for mechanical and cold sensitivity testing (sham treatment, repetitive PI tDCS) and were divided into another three groups for the immunohistochemistry experiments (sham treatment, single PI tDCS, repetitive PI tDCS). From 3 to 36 days following the spared nerve injury (SNI), mice underwent daily tDCS sessions for five consecutive days at an early and late stage of neuropathic pain, labelled Block I and Block II, respectively. Mechanical and cold sensitivity were assessed at one day before nerve injury, one day before the first tDCS treatment session in both blocks, and on defined days over a 21-day period following the final tDCS treatment session in each block. Motor function (open field test) and anxiety (elevated plus maze) were assessed separately in Block I and II, respectively. Aversiveness to mechanical stimulation with the place escape/avoidance paradigm test was assessed in both two blocks following the final repetitive tDCS treatment sessions (11 and 44 days following the SNI). For the study of the descending pathway, mechanical sensitivity was assessed one day before an acute intrathecal injection of naloxone or sterile saline on the fourth day post-tDCS (day 75 post-SNI) in Block III, and on the fifth day, 30 min following intrathecal injection, one mouse in the sham treatment group and two mice in the repetitive PI tDCS group were excluded before Block II and III due to the disconnection of the electrode. For immunohistochemistry experiments, animals received either the sham treatment, single PI tDCS, or repetitive PI tDCS over five days, were killed 1 h later by a high dose of isoflurane and immediately perfused with formalin fixative. The experimenters were blinded to the identity of mice being analyzed in behavioral and immunohistochemistry analyses. Behavioral testing was performed during the light (day) phase.

### 2.3. Electrode Implantation

**Figure 1:**
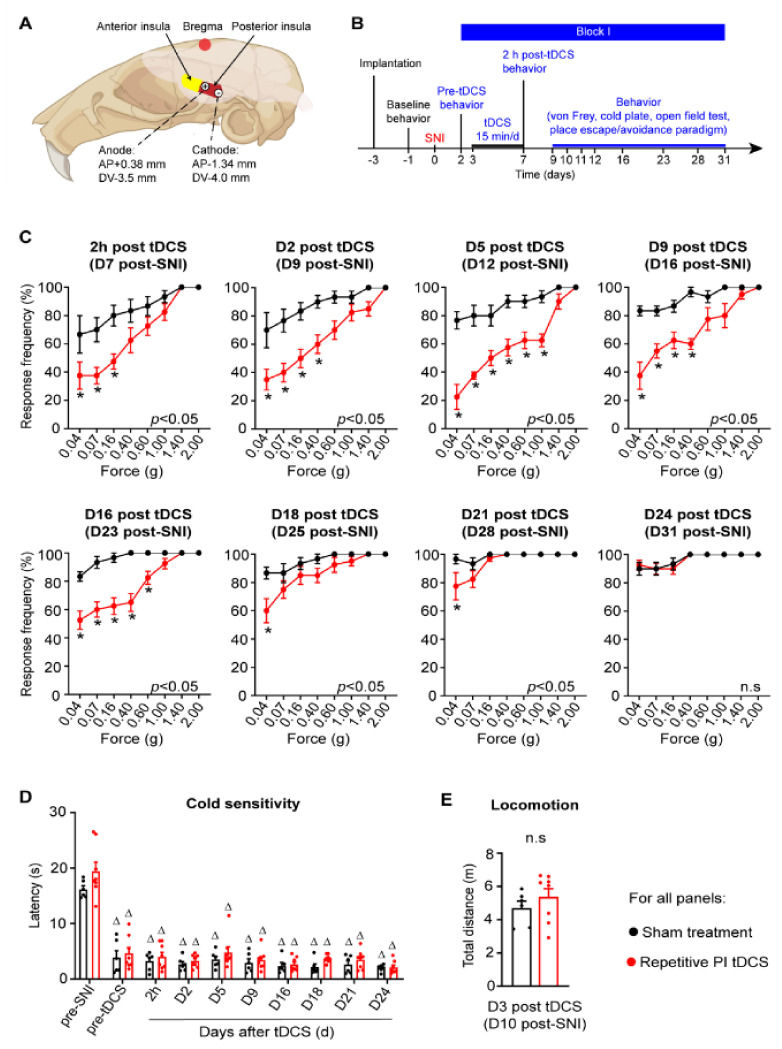
Effects of repetitive transcranial direct current stimulation applied to the posterior insula (PI tDCS) on mechanical sensitivity, cold sensitivity and locomotion in mice over the development of neuropathic pain. (**A**) Schematic illustration of the placement of electrodes over the temporal skull with anterio-posterior (AP), medio-lateral (ML) and depth coordinates relative to Bregma. (**B**) Schematic overview on the experimental plan and timeline involving the application of the first block of repetitive tDCS over 5 days in mice over early stages post spared nerve injury (SNI). (**C**) Behavioral analysis of mechanical sensitivity measured as rate of withdrawal responses to five applications of graded von Frey filaments (0.04 g to 2.0 g force) to the paw ipsilateral to the nerve injury in mice receiving PI tDCS or sham treatment without current application. (**D**) Behavioral analysis of cold sensitivity measured as latency to respond on a cold plate. (**E**) Analysis of mobility in an open field; *n* = 6 mice for the sham treatment group, *n* = 8 mice for the repetitive PI tDCS group; repeated measures including ANOVA with Sidak’s multiple comparisons test were performed; * *p* < 0.05 as compared to the sham treatment group. ^Δ^ *p* < 0.05 as compared to the pre-SNI value within each group; n.s. represents non-significant differences between two groups. Data are represented as mean ± standard error of the mean (S.E.M.).

Electrodes made of stainless steel with the size M1 × 10 mm were employed over the posterior part of the insular cortex on the right hemisphere (Figure 1A). Animals were anesthetized with an intraperitoneal injection of medetomidine (0.3 mg/kg; alvetra, Neumünster, Germany), fentanyl (0.01 mg/kg; Janssen-Cilag, Neuss, Germany) and midazolam (4 mg/kg; Hameln Pharma Plus, Hameln, Germany). The head of each mouse was fixed in a stereotaxic alignment system (David Kopf Instruments, Tujunga, CA, USA), and the skull was exposed by standard surgical procedures. The anodal electrode was centered at the anterior-posterior (AP) axis at +0.38 mm relative to Bregma and the mediolateral (ML) axis at +4.00 mm from the midline, and 3.50 mm in depth. The cathodal electrode was centered at AP: −1.34 mm, ML: +4.29 mm, and 4.00 mm in depth. Two holes were superficially made in the skull and the electrodes were mounted into holes separately and cemented onto the skull with three layers of dental cement (Paladur, Heraeus Kulzer, Germany). The electrodes were superficially fixed in the holes in order to avoid contact with the dura of the mouse brain. A mixture of naloxone (0.4 mg/kg; Inresa Arzneimittel, Freiburg, Germany), flumazenil (0.5 mg/kg; Fresenius, Bad Homburg, Germany), and antipamezol (2.5 mg/kg; Prodivet Pharmaceuticals, Belgium;) were injected intraperitoneally to end anaesthesia, and carprofen (5 mg/kg; Norbrook Laboratories, Newry, Northern Ireland) was given to protect against postoperative pain. The animals were placed on a warm heating plate until fully recovered. Coordinates for diverse brain regions in this study were based on the mouse brain atlas by Paxinos and Franklin [18].

### 2.4. Spared Nerve Injury (SNI)

After recovery from electrode implantation, animals were anesthetized again with the medetomidine/midazolam/fentanyl mixture (see above). As described previously [9,19], the sciatic nerve and its three branches (sural, common peroneal, and tibial) were exposed via an incision of the lateral thigh skin and a dissection of the biceps femoris muscle, and the common peroneal and tibial nerves were tightly ligated and cut distally; a 2–4 mm section was removed from the ligation. The sural nerve remained intact during surgery. 

### 2.5. Transcranial Current Stimulation (tDCS)

All animals received 5 treatment sessions per block (15 min per day over 5 consecutive days). Treatment was initiated at day 3, Day 36 and day 75 post-SNI in Block I, Block II and Block III, respectively. The tDCS protocol was adapted from previous tDCS studies [9,11,20,21,22,23]. Animals were anesthetized with 3% isoflurane, the head fixed via the nosepiece of the stereotaxic mask (RWD Life Science Company, China), and a light sedation maintained with 1% isoflurane. Tungsten wire electrodes attached to the anterior electrode (near to eye) serving as anode (+) and posterior electrode (near to ear) as cathode (−) (Figure 1A). Constant current at 50 μA was applied for 15 min via an A320 stimulus isolator (World Precision Instruments Inc., Sarasota, FL, USA). Animals in the sham treatment group underwent same procedure but without switching on the stimulator.

### 2.6. Behavioral Tests

Mice were acclimatized to the von Frey setup for 1 h the day before baseline and pre-tDCS test days as well as at 30 min before each testing session. Following a five consecutive tDCS treatment session, mechanical and cold sensitivity tests were performed 2 h after the final tDCS session, and then at 2 days, 5 days, 9 days, 16 days, 18 days and 21 days post tDCS, respectively, in both blocks. Mice were not acclimatized on the elevated plus maze, the open field arena, or the place escape/avoidance paradigm arena. A locomotion test in the open field was performed at 3 days post treatment in both blocks. Anxiety-like behavior in the elevated plus maze was tested at 3 days post treatment in Block II. The place escape/avoidance paradigm was analyzed at 4 days post treatment in both blocks. To study the effect of PI tDCS on descending pathways, a mechanical sensitivity test was performed before and 30 min after an acute non-invasive intrathecal injection of naloxone (0.4 μg/μL, 5 μL; Inresa Arzneimittel, Freiburg, Germany) or saline injection, which was given under 1% isoflurane anesthesia as previously described [24].

### 2.7. Von Frey Filaments (VF)

Mechanical sensitivity of the affected hind paw was tested on an elevated grid (Ugo Basile Inc., Gemonio, Italy) with manually repeated von Frey filaments application with increasing forces (0.04–2.00 g) [25]. Briefly, von Frey filaments were applied to the lateral plantar surface of the affected hind paw. Withdrawal frequencies were determined from five applications per filament, with a minimal interval of 30 s between applications. Paw lifting was defined as a positive response.

### 2.8. Cold Plate Test

Cold sensitivity was tested on a circular cold metal surface (5 °C, Hot/Cold Plate 35,100, Ugo Basile Inc., Gemonio, Italy) enclosed by a Perspex cylinder. Latency of the first nociceptive response (paw lifting, shaking, licking, or jumping) was monitored. A 30 s cut-off was used to prevent potential injury to the paws. Cold sensitivity was tested only once per testing session.

### 2.9. Mobility in an Open Field

Mobility was tested in a custom-made testing arena placed on the floor, with dimensions of 40 (length) × 40 (width) × 38 (height) cm. Mice were allowed to explore the arena freely for 10 min [26,27,28]. The experiment was video-recorded and tracked using ANY-maze software (Stoelting Co., Dublin, Ireland).

### 2.10. Elevated Plus-Maze (EPM) Test

Anxiety-related behavior was evaluated based on the cumulative exploration time spent by each mouse in the open zones of an EPM apparatus [29,30,31]. The maze consists of four arms, two open arms without walls and two arms enclosed by 15 cm high walls, each of which was 35 cm long, 5 cm wide, and raised 50 cm from the floor. The four arms met at a 5 × 5 cm central intersection. Mice were placed at the junction of the open and covered arms, facing away from an open arm, where the experimenter was positioned, and allowed to move freely for five minutes. Time spent in each zone was recorded and tracked using ANY-maze software (Version 7.1, Stoelting Co., Dublin, Ireland). 

### 2.11. Place Escape/Avoidance Paradigm (PEAP)

In order to assess the aversiveness of evoked mechanical stimulation in neuropathic animals, PEAP was performed four days after the last session of tDCS treatment in both blocks, and was modified from previous studies [9,11,32]. Briefly, animals were given a unilateral hind paw nociceptive stimulation and were placed in a 22 × 22 × 12 cm chamber atop a mesh floor. One half of the chamber is covered with white foil (light area) and the other half of the chamber is covered with black foil (dark area), connected to a 3.5 cm opening in the dividing wall. The behavior of the animal was typically assessed during a 30-min test, with the animal allowed unrestricted movement within the chamber and between the two sides. The first 5 min out of 30 min was defined as an unstimulated reference baseline. At 15 s intervals, a suprathreshold von Frey monofilament (0.07 g was applied in Block I, 0.16 g was applied in Block II) was used to stimulate the lateral plantar surface of a single hind paw. When the animal was located within the dark side of the chamber, the affected paw was stimulated, and the unaffected paw was stimulated while within the light side of the chamber. The entire period of testing was digitally recorded via a USB camera and analyzed by ANY-maze software (Version 7.1, Stoelting Co., Dublin, Ireland).

### 2.12. Intrathecal Injections

To study the effect of PI tDCS on descending pathways, von Frey baseline mechanical testing was performed 4 days after the last session of tDCS treatment (Block III), but before an acute intrathecal injection of naloxone (0.4 μg/μL, 5 μL; Inresa Arzneimittel, Freiburg, Germany) or saline injection, which was given under 1% isoflurane anesthesia as previously described [24]. This procedure involved locating the prominent spinous process of L6 with a gentle press and carefully inserting the needle (needle size, 31 G) between the grooves of the L5 and L6 vertebra. A tail flick during needle insertion indicates successful entry of the needle in the intradural space. Animals that did not display the tail flick were not used for the further experiment. Mechanical basal sensitivity was tested 4 days after the last session of the third round of tDCS treatment in Block III (day 75 post-SNI). Intrathecal injection was applied at the fifth day post-tDCS. Thirty minutes after the injection, the von Frey measurement was performed. The animals subsequently received an intrathecal injection of saline (if they had received naloxone previously) or naloxone (if treated with saline before) the next day. The experimenter taking the measurements was always blinded to both the identities of the animals (sham treatment or repetitive PI tDCS) and to the drug (saline or naloxone) that was injected.

### 2.13. Immunohistochemistry

Non-SNI mice were killed 1 h after either a single tDCS or sham and repetitive session in the first treatment block; SNI mice were killed 4 days after sham or repetitive session in the second treatment block. Animals were perfused transcardially with phosphate-buffer saline (PBS; pH 7.4) followed by 10 % formalin fixative solution (Merck, Darmstadt, Germany). Brains were sectioned coronally at 50 μm were stained as previous described [11] with primary anti-Fos (Rabbit-anti-Fos, 1:1000, Abcam, ab190289, Cambridge, UK) and secondary antibody (Donkey anti-rabbit Alexa 488, 1:700, Invitrogen, Carlsbad, CA, USA). Frozen sections at 25 µm thickness from spinal segments (L3–L5) were processed similarly with the exception that antigen retrieval was performed via incubation in 1 mM EDTA solution prior to the actual staining procedure. Unspecifc antigen blocking was achieved by incubation for 30 min in PBS-T containing 5% (*v*/*v*) donkey serum (Abcam, ab7475, Cambridge, UK). Rabbit-anti-Fos (1:1000, Abcam, ab190289, Cambridge, UK) was applied for 1 h at room temperature followed by 48 h at 4 °C. Secondary donkey anti-rabbit Alexa 488-conjugated antibody (1:700, Invitrogen, Carlsbad, CA, USA) diluted in blocking solution was employed, and nuclei were counterstained using Hoechst 33,342 (1:10,000, Molecular Probes, Eugene, OR, USA). 

### 2.14. Image Acquisition and Quantification

Labelled sections were imaged with a Leica TCS SP8 confocal microscope using sequential line scans at a pixel resolution of 1024 × 1024, with the pin hole set to unity. An immersion objective with correction collar (Leica, 20×/0.75, HC PL APO) was used for imaging either Fos-labelled section. A montage of confocal image stacks was acquired over a depth of 20 μm. The maximum z-projection brain images were applied for manual analysis by Fuji-Image J software (version 1.50b, National Institutes of Health, Bethesda, MD, USA), using a thresholding approach (threshold > 30, pixel^˄^2 size > 6, circularity 0.23–1.0) on 8-bit format images and data from the stereotaxic atlas [18] was used to define region of interest outlines anatomically according to corresponding reference sections. After manually counting, the results were further screened manually to exclude false positives. 

### 2.15. Statistical Analyses

A normal distribution of the data was verified in Prism (Version 8.0, Graphpad Software Inc., San Diego, CA, USA) using the D’Agostino-Pearson Omnibus K2 normality test, and all data is expressed as mean ± standard error of the mean (S.E.M). A statistical significance of difference was determined using a one-way ANOVA test, two-way ANOVA with post hoc Sidak’s test, Tukey’s tests enabling multiple comparisons, or a Mann-Whitney test using Prism. A *p*-value of < 0.05 was considered significant in all tests.

## 3. Results

### 3.1. Impact of tDCS of the Posterior Insula (PI) on the Development of Neuropathic Hypersensitivity

One of the caveats of transcranial electrical stimulation is that the current spreads broadly beyond the target neocortical area to other cortices. In this study, we therefore placed both anodal and cathodal electrodes over the PI, such that current would only flow over the PI and minimize spread to the neighbouring regions (schematic in Figure 1A). We then studied the sensitivity to mechanical and cold stimuli applied to the contralateral paw, which also underwent spared nerve injury (SNI) in a very well-established model of neuropathic pain. In the first set of experiments, we applied one block of tDCS at 0.35 mA current (repetitive PI tDCS group) or 0 mA current (sham treatment group) at 5 daily repetitions for 15 min over a period of 5 days in the first week post-SNI (Block I; see experimental scheme in Figure 1B). As known previously, graded strengths of mechanical von Frey stimuli applied to the plantar hindpaw led to graded withdrawal responses (see stimulus-response curves in Supplementary Figure S1A). SNI induced a significantly higher response rate to stimuli that are non-noxious under control (baseline) conditions (mechanical allodynia) by day 2 post-nerve injury, which continued to rise over several weeks post-SNI (black symbols in Figure 1C). In comparison with mice receiving sham stimulation, mice which received Block 1 PI tDCS developed mechanical hypersensitivity to a dramatically lower extent (red symbols in Figure 1C). Importantly, PI tDCS-induced protection against the development of mechanical allodynia was evident when tested directly after the last tDCS treatment in Block I on day 7 post-SNI, and it was still very high until 16 days post-tDCS (day 23 post-SNI) and still significant until 21 days post-tDCS. As of day 24 post-stimulation, PI tDCS SNI mice were indistinguishable from SNI mice that received sham stimulation treatment. 

Surprisingly, despite this strong protection against the development of mechanical hypersensitivity, hypersensitivity to cold, which is another hallmark of neuropathic pain [1], developed to a similar extent in mice receiving PI tDCS or sham stimulation (Figure 1D). Furthermore, locomotion and overall mobility in an open field setting was not affected by PI tDCS (Figure 1E). These results thus indicate that performing a 5-day regimen of tDCS over the PI following a major nerve injury can significantly reduce the development of debilitating mechanical allodynia for 3 weeks post-injury without altering mobility.

### 3.2. Reversal of Established Neuropathic Mechanical and Cold Allodynia by a Second Regimen of PI tDCS

**Figure 2:**
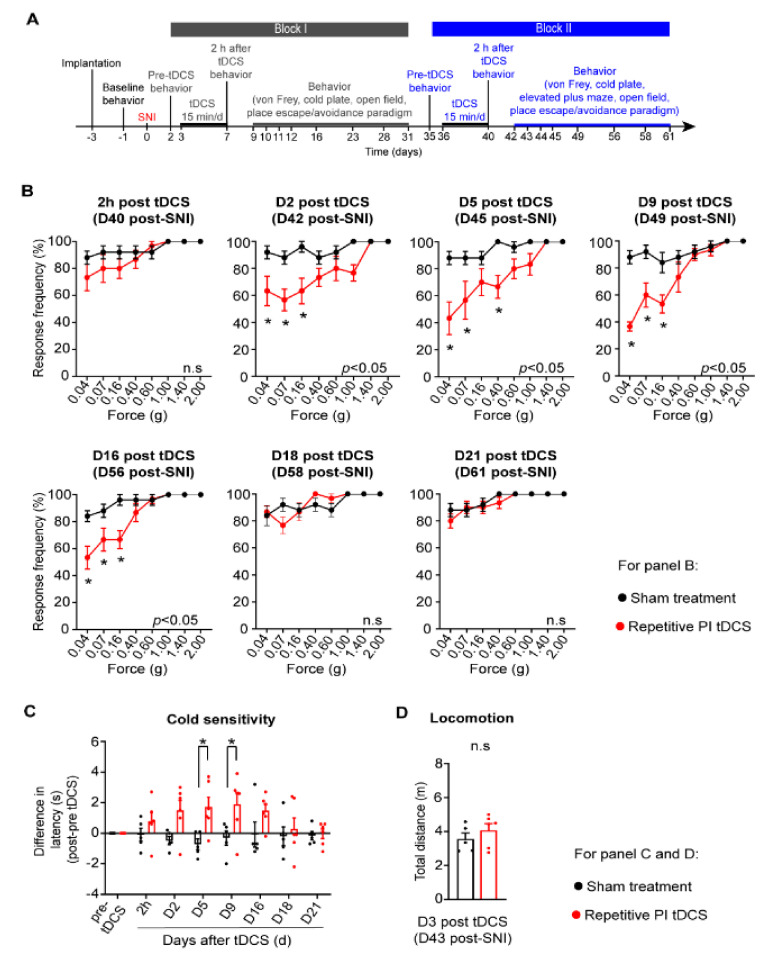
Reversal of established mechanical allodynia and cold allodynia over the late stages of neuropathic pain post-nerve injury by repetitive PI tDCS. (**A**) Schematic overview of experimental plan involving application of repetitive tDCS (Block II) several weeks after nerve injury. (**B**) Behavioral analysis of mechanical hypersensitivity to graded von Frey filaments over 40–61 days post-SNI. (**C**) Behavioral analysis of cold hypersensitivity to 5 °C in the same groups of mice. (**D**) Effect of Block II PI tDCS on mobility in an open field; *n* = 5 mice for the sham treatment group, *n* = 6 mice for the repetitive PI tDCS group; repeated measures ANOVA with Sidak’s multiple comparisons test was performed; * *p* < 0.05 as compared to the sham treatment group. n.s. represents non-significant differences between two groups. Data are represented as mean ± S.E.M.

In the clinical setting, it is common that neuropathic pain becomes chronic and patients seek therapeutic help when neuropathic pain is already established as a chronic disorder [1]. We therefore tested the therapeutic impact of a second regimen of tDCS starting at day 36 after nerve injury (Block II, see experimental scheme in Figure 2A). Even at this chronic stage of the neuropathic pain disorder, PI tDCS led to a significant and robust decrease in mechanical allodynia until day 16 after block II stimulation (day 56 post-SNI). Thus, chronically established neuropathic mechanical allodynia can be reversed by repetitive PI tDCS. Remarkably, we also observed that this second regimen also significantly reduced chronically established cold allodynia (Figure 2B). Repeating the tDCS of the PI did not lead to any deleterious effects on overall well-being, and mice showed normal activity and mobility (Figure 2C). 

### 3.3. Impact of PI tDCS on Negative Affect and Anxiety

**Figure 3:**
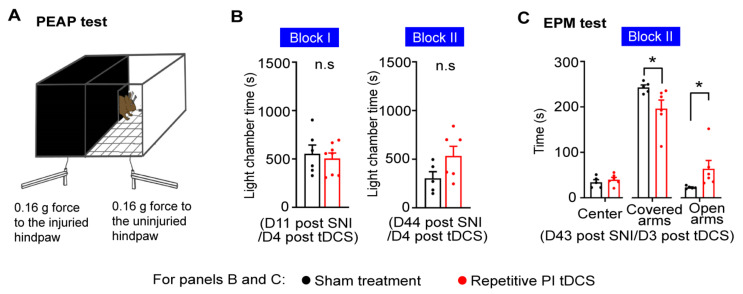
Impact of repetitive PI tDCS on the aversive component of neuropathic pain and pain-related anxiety-like behavior. (**A**) Scheme of the place escape avoidance paradigm (PEAP test), which tests aversion to low intensity von Frey force in neuropathic mice with mechanical allodynia. (**B**) Quantitative summary of time spent in the light chamber (i.e., avoiding the dark side of the chamber). (**C**) Analysis of anxiety-like behavior in neuropathic mice, demonstrated by more time spent in closed arms than in open arms of the elevated maze. For panel (**B**), Block I: *n* = 6 in sham treatment group and *n* = 8 in PI tDCS group, in panels (**B**,**C**), Block II: *n* = 5 in sham treatment group and *n* = 6 in PI tDCS group; repeated ANOVA measurements with Sidak’s multiple comparisons test and the Mann-Whitney test were performed; * *p* < 0.05 as compared to the sham treatment group. n.s.: non-significant. Data are represented as mean ± S.E.M.

An important component of pain is negative affect, which is challenging to study in rodent behavior. Testing conditioned preference or avoidance behaviors [33] are difficult in the setting of this study, since tDCS treatment essentially extends over several days and unfolds its beneficial effects over the weeks after completing the last treatment. We therefore tested voluntary avoidance behaviors towards stimuli that are innocuous in control conditions but become noxious following nerve injury using a paradigm of place escape avoidance (PEAP) which we have established and reported on previously [9,11]. Because mice prefer a dark environment, using a light-dark box, we paired a dark chamber with the application of 0.07–0.16 g von Frey mechanical stimuli on the paw ipsilateral to nerve injury and application of the same stimuli to the contralateral unaffected paw in the bright chamber (Figure 3A). Avoidance to the stimulus evoking mechanical allodynia, which is indicative of the aversive, negative affective component of pain, was not significantly altered after application of either Block I or Block II PI tDCS regimens (Figure 3B).

Because chronic pain patients also frequently develop anxiety and fear when neuropathic pain becomes chronic, we also tested behaviors in an elevated plus maze (EPM) apparatus. Neuropathic mice receiving PI tDCS showed significantly reduced time in the covered arms of the maze and spent significantly more time in the open arms (Figure 3C), suggesting a reduction in anxiety. Thus, repetitive neurostimulation of the PI did not alleviate the negative affective component of pain but reduced anxiety associated with neuropathic pain.

### 3.4. Contribution of Descending Inhibition to the Antiallodynic Effects of PI tDCS

**Figure 4:**
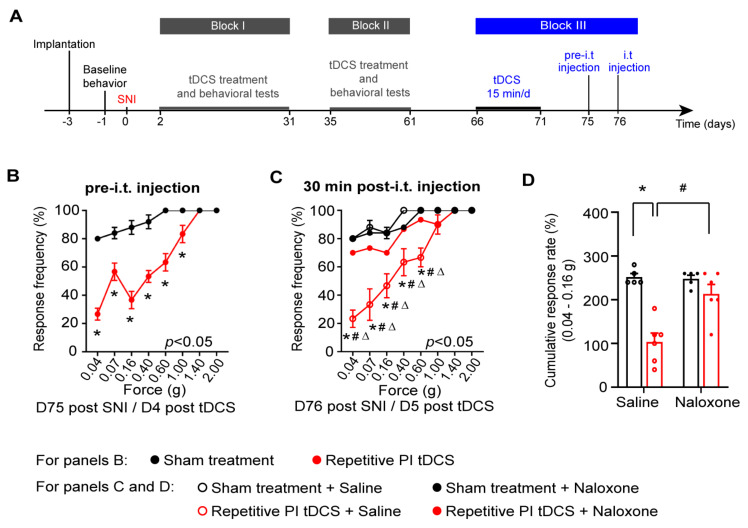
Anti-allodynic effect of repetitive PI tDCS requires spinal opioidergic signaling. (**A**) Schematic overview of testing the effect of intrathecal (i.t.) injection of the opioid-antagonist naloxone or saline (control) on suppression of mechanical allodynia over chronic stages of neuropathic pain. (**B**) Comparison of neuropathic mechanical allodynia between the groups of sham treatment and PI tDCS 5 days after the last session of the treatment prior to i.t. injection of naloxone or saline. (**C**,**D**) In the same mice, analysis of neuropathic mechanical allodynia after i.t. injection of naloxone or saline, showing naloxone-induced reversal of the antiallodynic effect of PI tDCS. Panel (**C**) shows the stimulus response curve to graded von Frey filaments and panel (**D**) shows cumulative responses to filaments exerting low forces that are non-noxious in control conditions and elicit allodynia in neuropathic conditions; *n* = 5 for the sham treatment group and *n* = 6 for the repetitive PI tDCS group; repeated measures ANOVA with Sidak’s multiple comparisons test was performed; * *p* < 0.05 as compared to the sham treatment group. # *p* < 0.05, Δ *p* < 0.05 as compared to the corresponding control group. n.s.: non-significant. Data are represented as mean ± S.E.M.

Given that the tDCS of the PI affected the nociceptive withdrawal behavior in neuropathic mice, and because the PI has been reported to modulate brainstem centers engaged in descending modulation of spinally-evoked nociceptive behaviors, we tested the role of descending modulation in the antinociceptive effects of PI tDCS. Opioidergic control in the brainstem engages descending serotonergic and noradrenergic pathways, which can, in turn, recruit spinal enkephelinergic neurons in the spinal cord to inhibit nociceptive transmission [34,35]. In the cohort of mice represented in Figure 2, we therefore addressed whether tonic opioidergic signaling is contributing to tDCS-induced analgesia by intrathecally (spinally) injecting the opioid receptor blocker, naloxone, or saline (control), in conjunction with a third block of tDCS (experimental scheme in Figure 4A). These experiments revealed two insights: One, repeating another block of PI tDCS at this very later stage of chronically established neuropathic pain again robustly reduced mechanical allodynia (Figure 4B). Secondly, blocking opioidergic signalling in the spinal cord with naloxone completely blocked the beneficial effect of PI tDCS on mechanical allodynia, while it was preserved upon intrathecal saline application (Figure 4C,D). These results demonstrate that the posterior insular engages descending modulatory circuits to inhibit mechanical hypersensitivity neuropathic pain.

### 3.5. Activity Mapping in the Insula and Pain-Related Brain Regions following PI tDCS

**Figure 5:**
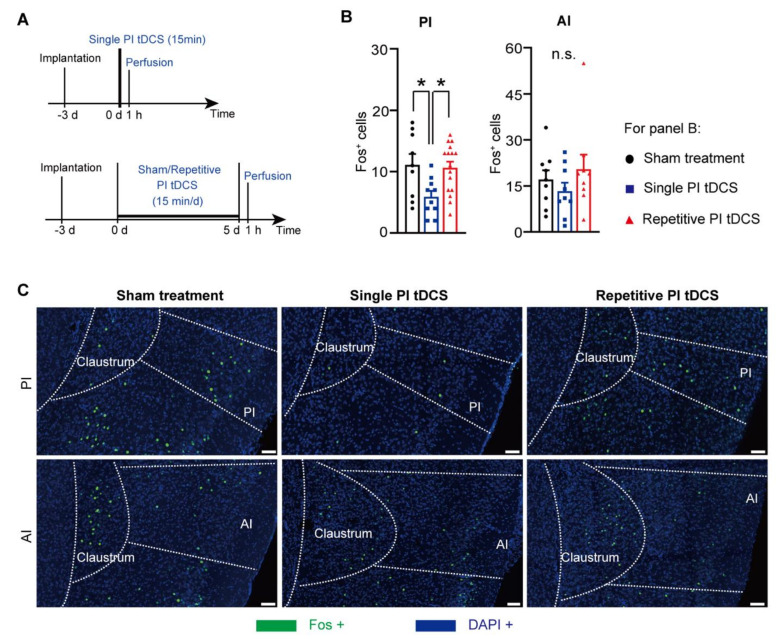
Immunohistochemical analysis of Fos, the product of the activity marker gene *c-Fos* in the anterior insula (AI) and PI following stimulation of PI. (**A**) Experimental scheme for testing the impact of a single PI tDCS session or repetitive stimulation over five consecutive days compared to sham treatment. (**B**) Quantitative analysis of number of Fos-expressing cells per region of interest in the PI and AI. (**C**) Typical examples of Fos immunoreactivity with DAPI counterstaining over the PI and AI; scale bars represent 100 μm; *n* = 9 sections from three mice in the sham treatment group, *n* = 10 sections from four mice in the single PI tDCS group and *n* = 16 sections from four mice in the repetitive PI tDCS group; repeated ANOVA with Sidak’s multiple comparisons tests were performed; * *p* < 0.05 as compared to the corresponding control group. n.s.: non-significant. Data are represented as mean ± S.E.M.

We then sought to address how tDCS over the PI leads to changes in activity patterns within the insular cortex and in diverse areas of the brain that are associated with pain perception and pain modulation. Towards this end, we employed immunohistochemistry against c-Fos, a surrogate marker for active neurons and test the impact of a single stimulation versus the repetitive regime of five consecutive simulations that was employed in the study (see experimental scheme in Figure 5A), as described previously [11]. Despite its close proximity, Fos expression in the anterior insula was not affected by PI tDCS (Figure 5B,C), demonstrating that the flow of the current was indeed restricted to the PI. Surprisingly, we observed that a single stimulus led to a significant decrease in the number of Fos-positive neurons in the PI (Figure 5B,C), suggesting that the flow of the current within the PI depresses its activity locally instead of increasing it. However, upon repetitive stimulation, this effect was lost. 

**Figure 6:**
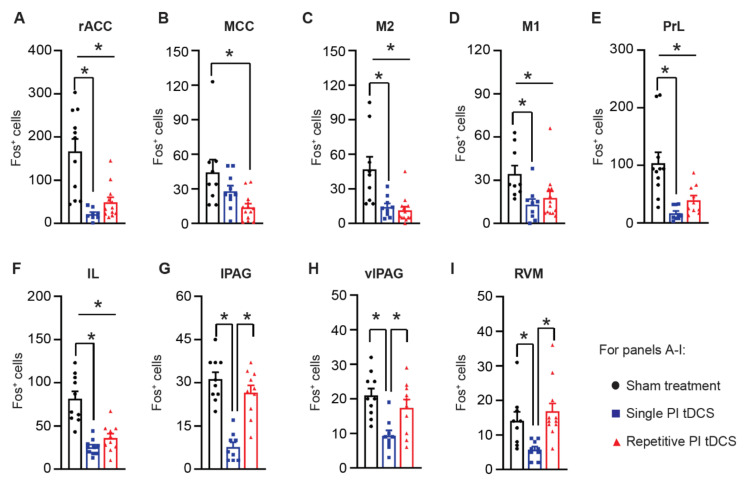
Immunohistochemical analysis of the surrogate marker for neuronal activity, Fos, in diverse brain regions following tDCS stimulation of PI in the form of either a single PI tDCS session or repetitive stimulation over five consecutive days compared to sham treatment. (**A**–**I**) Quantitative analysis of a number of Fos-expressing cells per region of interest. *n* = 9 sections from three mice in the sham treatment group, *n* = 10 sections from four mice in the single PI tDCS group, and *n* = 16 sections from four mice in the repetitive PI tDCS group; repeated ANOVA with Sidak’s multiple comparisons tests were performed; * *p* < 0.05 as compared to the corresponding control group. n.s.: non-significant. Data are represented as mean ± S.E.M. Abbreviations: rostral anterior cingulate cortex (rACC), midcingulate cortex (MCC), primary and secondary motor cortex (M1 and M2), prelimbic cortex (PrL), infralimbic cortex (IL), lateral periaqueductal grey (lPAG), ventrolateral periaqueductal grey (vlPAG), and rostral ventromedial medulla (RVM).

The posterior insula is well connected to other diverse neocortical areas [36], and we therefore tested how they change in activity upon single or repetitive tDCS of the PI. Several regions within the prefrontal cortex, including the rostral anterior cingulate cortex (rACC), the prelimbic cortex (PrL) and infralimbic cortex (IL), and the primary and secondary motor cortex (M1 and M2) showed a significant suppression of activity upon a single tDCS application to the PI, which was also maintained upon repetitive stimulation (examples in Figure 6A and quantitative summary in Figure 6B). The mid-cingulate cortex (MCC) showed a decrease in Fos-positive active neurons only after repetitive PI tDCS. Surprisingly, although key brainstem centers involved in descending modulation, such as the rostroventral medulla (RVM) and the lateral and ventrolateral periaqueductal grey (lPAG and vlPAG), showed an initial decrease in activity marker expression after a single tDCS of the PI; repetitive stimulation led to normalization of their activity back to baseline (control) levels (Figure 6A,B). Thus, while a number of brain regions initially showed decreased activity upon tDCS of the PI, several neocortical areas involved in pain modulation demonstrated a persistent suppression of baseline activity levels following repetitive PI tDCS, indicating a major remodeling of pain-related networks in the brain. 

**Figure 7:**
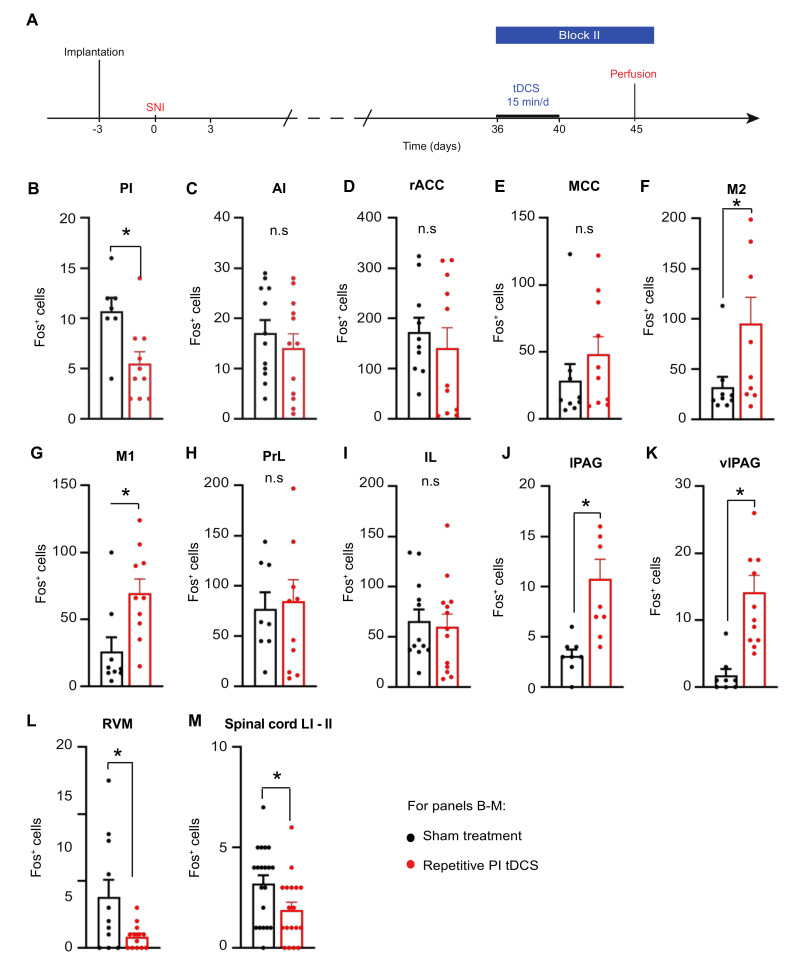
Immunohistochemical analysis of Fos in diverse brain regions following repetitive tDCS or sham stimulation of the PI in mice with neuropathic pain. Shown are the experimental scheme of tDCS or sham stimulation after SNI (**A**) and quantitative summary of Fos-positive cells in diverse brain regions (**B**–**M**). *n* = 6–12 sections from two to four mice for each area; an unpaired t test was performed; * *p* < 0.05 as compared to the corresponding control group. n.s.: non-significant. Additional abbreviations: LI-LII: spinal superficial laminae I and II.

Finally, we addressed whether repetitive PI tDCS affects the activity of brain regions differently in mice with neuropathic pain (Figure 7A), since nerve injury itself leads to a major remodeling of the brain circuitry. Indeed, in contrast to uninjured mice, neuropathic mice demonstrated a decrease in Fos-expressing neurons of the PI following repetitive PI tDCS as compared to sham-treated neuropathic mice (Figure 7B), while AI activity remained unchanged (Figure 7C). Similarly, unlike uninjured mice, Fos-positive cells were not reduced by repetitive PI tDCS in the rACC, MCC, PrL and IL (Figure 7D,E,H,I). While repetitive PI tDCS suppressed activity of the M1 and M2 in uninjured mice, these regions showed higher Fos-positive cells in neuropathic mice (Figure 7F,G). Finally, and most importantly, repetitive PI tDCS strongly influenced the activity of descending modulatory centers by increasing Fos-positive cells in the lPAG and vlPAG (Figure 7J,K) and decreasing Fos-positive cells in the RVM and the superficial spinal laminae (Figure 7L,M). These data thus strongly support the point that repetitive PI tDCS acts by promoting descending inhibition and suppressing descending facilitation.

## 4. Discussion

Non-invasive brain stimulation is increasingly gaining value as a therapy for chronic pain that is refractory to drug treatments [8]. While there is consensus that this new line of therapy should be increasingly tested and developed for routine clinical practice, there are major hurdles that need to be overcome, such as the high degree of variability in efficacy across clinical cohorts and types of chronic pain, the tradeoff between efficacy and safety, the need for optimization and standardization of treatment types and regimens, and the lack of mechanistic insights, amongst others [4,5]. Addressing these in the human context is challenging, costly and, not least, limited in the ability to perform invasive interventions and study mechanisms [37]. Several studies have demonstrated the utility of animal models in compensating for these limitations, but were so far limited to neurostimulation of the motor cortex and prefrontal cortex [9,10,11]. The value of this study is: (i) it is the first study, to our knowledge, testing direct neurostimulation of the insular cortex; (ii) the study tests a new approach for limiting the spread of activity to other cerebral cortical domains; (iii) the study addresses the changes in insular activity that are associated with pain relief; (iv) the study addresses mechanisms and circuitry downstream of the insula in modulating pain; and, finally, (v) the study uncovers the tremendous therapeutic potential of targeting the insular cortex in neurostimulation approaches in reversing some of the debilitating symptoms of chronically established neuropathic pain.

Until we performed the experiments and evaluated the data, how tDCS of the insula would affect neuropathic pain was completely open, and potential scenarios reflecting both the exacerbation of pain or the inhibition of pain were equally possible. This is because previous reports on insula activation via ictal discharges suggest that it can evoke pain [12], and findings in animal models also support a pro-nociceptive role for the insula [38]. The synaptic potentiation in the insula has been reported following nerve injury [17]. Therefore, activation of the insula would be expected to excarbate neuropathic pain-like behaviors. On the other hand, however, tDCS-induced activation of other brain regions that are typically activated during pain and are linked to nociceptive sensitization, such as the prelimbic and cingulate cortices, can paradoxically lead to analgesic effects [2]. Here, we observed a profound suppression of sensory hypersensitivity in nerve-injured mice upon PI tDCS, and given that we have previously performed tDCS on the prefrontal cortex and motor cortex, we noted that the magnitude of antinociceptive effects was comparatively stronger with PI tDCS. The antinociceptive effects of PI tDCS were not attributable to changes in motor function since we observed changes in paw withdrawal selectively to some modalities of nociception, and locomotion was not affected. 

Neurostimulation does not necessarily involve the activation of neurons; rather, the change in activity is determined by anodal or cathodal modes of stimulation and the stimulation parameters in terms of intensity, frequency, and rhythm, amongst others [6]. Anodal stimulation has been linked to the activation and cathodal to the inhibition of neuronal activity, and the design of tDCS in previous studies involved the flow of current across large parts of the cerebral cortex [6]. While this may have contributed to the analgesia seen with cortical neurostimulation, activating large parts of the brain is undesired given the massive functional diversity and significance of the cortex in brain functions. Here, in an attempt to restrict neurostimulation to the insula, we positioned both electrodes in the PI relative to each other along the anterior-posterior axis. Although we cannot control for the path of the current flow, the Fos activation pattern demonstrates that the current flow between the electrodes led to a reasonably selective manipulation of the PI area located between the two electrode poles. This setup is not dissimilar to bipolar stimulation configurations employed in acute brain slice recordings or in vivo bipolar microstimulation, and our analyses now demonstrate that this could be useful for delineating the impact of individual cerebral cortical domains, which is important given that domains located in close proximity can demonstrate highly divergent functions. 

Using this setup, we observed that single sessions of tDCS actually led to an acute inhibition of the PI, which is different from the previous studies on anodal stimulation of the motor cortex [9] and the prefrontal cortex [11]. Similar to those previous studies, our analyses also revealed that repeating tDCS sessions over five days, which evoked profound antinociceptive effects, led to a normalization of activity in the PI. Importantly, however, under neuropathic conditions, repetitive PI tDCS strongly suppressed indicators of activity in the PI, thus revealing that adaptive mechanisms come into play. Such adaptive mechanisms may span diverse mechanistic levels, including synaptic changes in both structure and function, changes in excitation-inhibition balance alterations in cortical columns of the PI via cellular changes in excitatory and inhibitory neuronal cell types, plasticity of connectivity and oscillatory rhythmic activity, amongst others. Uncovering their nature in future studies will be of critical value. 

Our analyses here provided two insights into changes in connectivity and activity downstream of the PI that are elicited by repetitive tDCS. Firstly, we observed that descending inhibitory systems which lead to spinal antinociception by evoking the local release of endogenous opioids in the spinal cord are necessarily involved since the antiallodynic effect of repetitive PI tDCS was blocked by the spinal application of the opioid antagonist naloxone. Bulbospinal pathways modulating spinal nociception can be both facilitatory and inhibitory in nature, and neuropathic hypersensitivity has been linked to a dominance of descending facilitation [34,35]. Our previous work has demonstrated that the functional connectivity between the PI and the RVM can recruit descending serotonergic facilitatory pathways [38]. Here, although repetitive PI tDCS did not alter activity of the RVM in uninjured mice, it strongly suppressed activity in the RVM of neuropathic mice. In line with this observation, the activation of the superficial spinal laminae was also suppressed by repetitive PI tDCS. These observations, coupled with the finding that the activity of the vlPAG and the lPAG was enhanced, suggests that repetitive PI tDCS restores the balance between descending inhibition and descending facilitation that is disturbed in neuropathic conditions, and thus overcomes the deficits in spinal inhibition in neuropathic pain. Furthermore, the observation that the application of repetitive PI tDCS enhances activity in the M1 cortex in neuropathic mice, but not in uninjured mice, implicates the recruitment of the M1, the stimulation of which by TMS or tDCS is known to relieve neuropathic pain [8,9,10]. Future studies functionally dissecting the interplay between diverse cerebral cortical domains in modulation of pain will provide valuable insights into exploiting these aspects of network plasticity towards long-term pain relief.

One noteworthy feature of the phenotypic consequences of repetitive PI tDCS is that we observed a robust effect on the sensory-discriminative component of neuropathic pain, but not on pain-related aversion and negative affect. This may reflect and indeed contribute functional experimental evidence for a functional dichotomy proposed by human brain imaging studies on nociceptive features of pain being encoded in the PI and affective dimensions of pain in the AI [14]. This is supported by connectivity analyses showing that the PI mainly receives nociceptive and thermoceptive information from the somatosensory thalamic nuclei [39]. In contrast, the anterior insula is implicated in the regulation of physiological changes associated with emotional states, which is consistent with its connectivity with multiple limbic sites involved in the affective aspects of pain, including the prefrontal cortex, pregenual cingulate cortex, the medial thalamic nucleus, and the amygdala [39] The posterior insula has been predicted to be a detector of the intensity of pain since its activation was found to be proportional to the intensity of a noxious stimulus independent of its quality in human experiments [13]. Taken together with this literature, our findings would predict that extending our PI tDCS setup to include the AI may enable targeting the emotional-affective dimension of pain by inhibiting the AI in addition to inhibiting allodynia via the PI.

Finally, the remarkably long duration of suppression of neuropathic allodynia upon PI tDCS and the reinstatement of this clinically relevant analgesic effect via multiple repetitions of the treatment cycle at chronic stages of neuropathic pain indicate an excellent basis for therapeutically developing neurostimulation focused on the insula. This study demonstrates that rodent models of human pain disorders can support developing novel neuromodulation techniques via a mechanism-based approach and will provide impetus for working out safe and effective parameters for clinical trials in humans.

## Data Availability

All raw data are included in the figures in form of individual data points in dot blots.

## Supplementary Materials

The following supporting information can be downloaded at: https://www.mdpi.com/article/10.3390/cells11203303/s1, Figure S1: Baseline mechanical sensitivity in treatment groups prior to PI tDCS procedure; Figure S2: Examples of Fos expression in uninjured mice with repetitive or single PI tDCS, Figure S3: Examples of Fos expression in mice with nerve injury with repetitive PI tDCS.
