# Supplementary material for: Prolonged Suppression of Neuropathic Hypersensitivity upon Neurostimulation of the Posterior Insula in Mice
Source: Cells. 2022 Oct 20;11(20):3303. doi: 10.3390/cells11203303 (PMC9600702; doi:10.3390/cells11203303)
Supplement: Supplementary file 1 [file cells-11-03303-s001.zip › cells-1864929-supplementary.pdf]

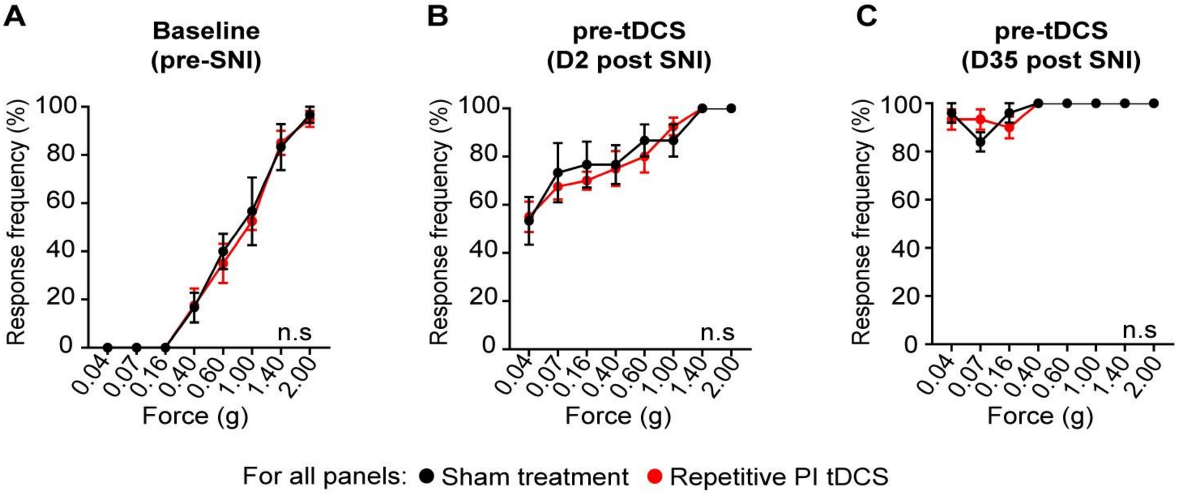

Figure S1. Baseline mechanical sensitivity in treatment groups prior to PI tDCS procedure. (A) Analysis of mechanical sensitivity to graded von Frey in the sham treatment group or the repetitive PI tDCS group before spared nerve injury (SNI). (B, C) Analysis of von Frey mechanical sensitivity in the sham treatment group or the repetitive PI tDCS group at 2 or 35 days after nerve injury but before tDCS treatment. In panels A and B, n = 6 mice for the sham treatment group and n = 8 mice for the repetitive PI tDCS group. In panel C, n = 5 mice for the sham treatment group and n = 6 mice for the repetitive PI tDCS group. Repeated measures ANOVA with post-hoc Tukey's multiple comparisons test was performed. n.s. represents non-significant differences between two groups. Data are represented as mean ± S.E.M.

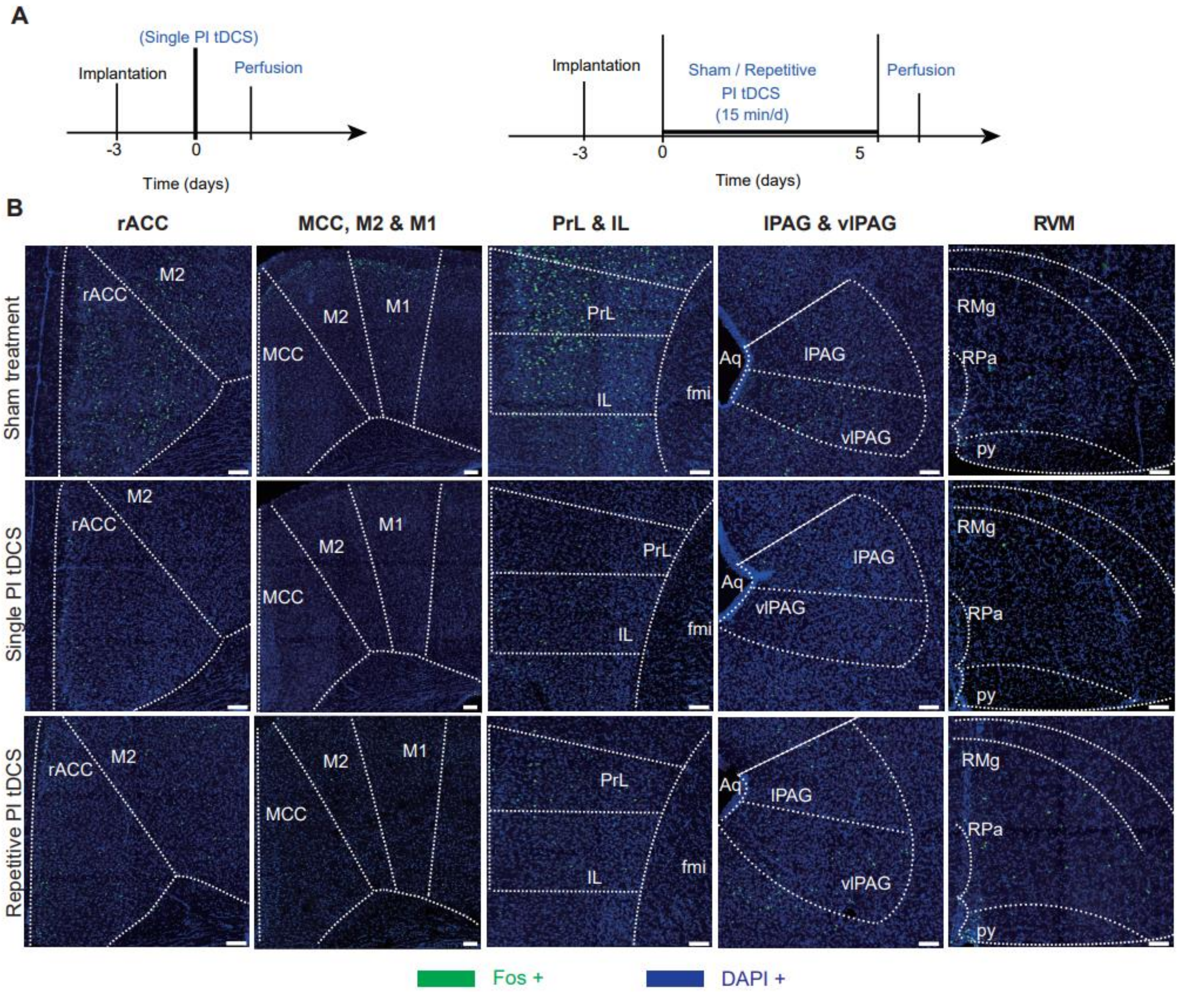

Figure S2. Examples of immunohistochemical analysis of the surrogate marker for neuronal activity, Fos, in diverse brain regions following tDCS stimulation of PI in form of either a single PI tDCS session or repetitive stimulation over 5 consecutive days compared to sham stimulation. Shown is the experimental scheme (**A**) and typical examples of Fos immunoreactivity with DAPI counterstaining (**B**). Abbreviations: rostral anterior cingulate cortex (rACC), midcingulate cortex (MCC), primary and secondary motor cortex (M1 and M2), prelimbic cortex (PrL), infralimbic cortex (IL), lateral periaqueductal grey (lPAG), ventrolateral periaqueductal grey (vlPAG), and rostral ventromedial medulla (RVM). Scale bar = 100 µm.

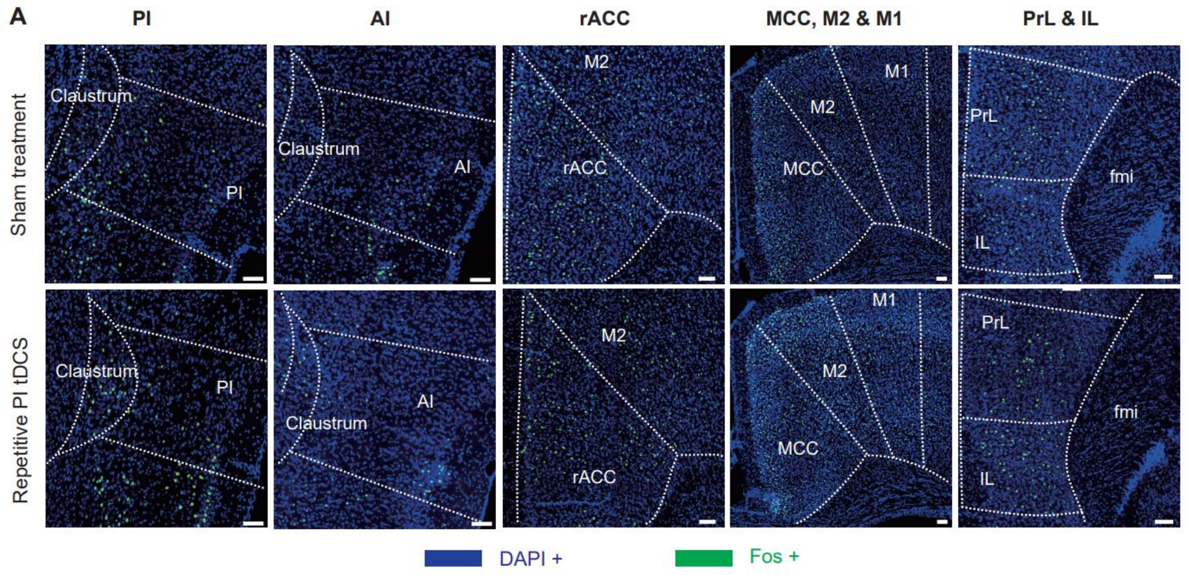

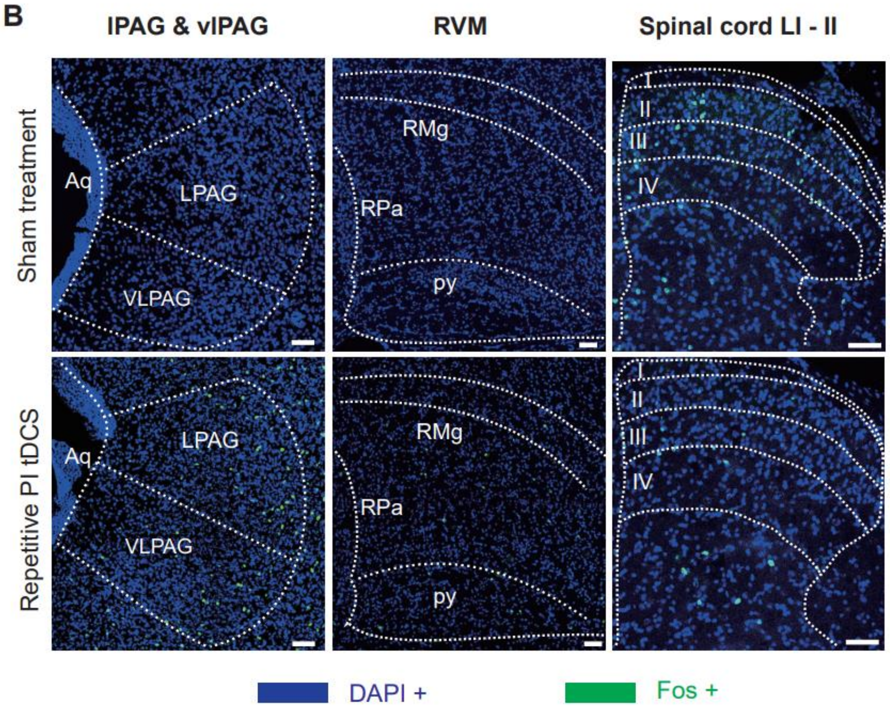

Figure S3. Examples of immunohistochemical analysis of Fos in diverse brain regions following repetitive tDCS or sham stimulation of the PI in mice with neuropathic pain. Shown are the experimental scheme of tDCS or sham stimulation after SNI (**A**) and typical examples of Fos immunoreactivity with DAPI counterstaining (**B**,**C**). Scale bar = 100 µm.
